# LAY PERSPECTIVES ON SUCCESSFUL AGING IN ROMANIA

**DOI:** 10.1093/geroni/igac059.2170

**Published:** 2022-12-20

**Authors:** Angélique Roquet, Mariana Barbut, Cornelia Pocnet, Daniela Jopp

**Affiliations:** University of Lausanne, Lausanne, Vaud, Switzerland; Lausanne University Hospital, Lausanne, Vaud, Switzerland; lausanne university, Lausanne, Vaud, Switzerland; University of Lausanne, Lausanne, Vaud, Switzerland

## Abstract

Successful aging is a topic of world-wide interest, yet much work focuses on specific and highly industrialized countries, such as the USA. The present study investigated lay perspectives of the concept of successful aging in young, middle-aged, and older adults from Romania. Ninety-three participants aged between 20 and 84 years were asked about definitions and determinants of successful aging. Based on a previous study, codes were developed to capture common themes among the answers, resulting in 14 categories. Participants mentioned four themes on average to describe successful aging, which varied by sociodemographic, health and psychological variables: individuals of younger age, with higher levels of education, in better health and those more satisfied with their aging mentioned more themes. Regarding specific topics, Social resources and Health were mentioned most frequently, followed by Quality of life, Financial situation, and Attitudes/Mechanisms. Young adults were more likely to mention Social resources, Success/Respect, and Aging themes, while older adults mentioned more often Meaning of life themes. Finally, we found that the themes mentioned were related to self-perception of aging, particularly among older adults: Older adults more satisfied and with positive views own aging were more likely to mention Social resources, Independence, and Attitudes/Mechanisms themes, while those with negative views on aging were more likely to mention Micro-environment themes. In sum, findings support the multidimensionality of lay perspectives of successful aging and offer insights on the understanding of successful aging in Eastern Europe, including topics and links to general and personal aging experiences.

